# Foley catheter balloon tamponade for actively bleeding wounds following penetrating neck injury: a systematic review and meta-analysis

**DOI:** 10.1308/rcsann.2025.0107

**Published:** 2025-12-15

**Authors:** LTP Tan, CYJ Lim, CX Li, V Kong, D Lee, J Ahn, D Wineberg, R Crawford, N Laher, G Oosthuizen, D Clarke

**Affiliations:** ^1^ University of Auckland, New Zealand; ^2^ Auckland City Hospital, New Zealand; ^3^ Chris Hani Baragwanath Hospital, Johannesburg, South Africa; ^4^ University of the Witwatersrand, Johannesburg, South Africa; ^5^ Tygerberg Hospital, Cape Town, South Africa; ^6^ Stellenbosch University, Cape Town, South Africa; ^7^ University of KwaZulu-Natal, Durban, South Africa

**Keywords:** Foley catheter, Balloon tamponade, Penetrating neck injury, Trauma

## Abstract

**Introduction:**

This study evaluated the efficacy and safety of Foley catheter balloon tamponade (FCBT) for actively bleeding penetrating neck injuries (PNI), focusing on rates of haemostatic success, subsequent surgical exploration, morbidity and mortality associated with FCBT in PNI.

**Methods:**

A systematic search of Medline/PubMed, Embase, CINAHL and the Cochrane Library was conducted to 29 May 2025. Observational studies reporting FCBT outcomes in actively bleeding PNI were included. Pooled proportions were calculated with random effects models using Freeman–Tukey double-arcsine transformation; heterogeneity was explored with subgroup analysis and meta-regression. Study quality was assessed using the ROBINS-I tool.

**Findings:**

Nine studies (1,658 participants) were included. FCBT was deployed in 236 cases, yielding a pooled rate of use of 27.85% (95% confidence interval [CI] 2.27 to 64.60, *I*^2^ = 97%, *p* < 0.01). Primary haemostasis was achieved in 62.50% to 100% across studies, with most series reporting success rates exceeding 80.00%. In total, 53.47% (95% CI 16.97 to 88.27, *I*^2^ = 61%, *p* = 0.0360) underwent surgical exploration, most often for major vascular injury or rebleeding at removal. Pooled morbidity and mortality were 11.70% (95% CI 0.00 to 50.47) and 6.30% (95% CI 0.00 to 19.97), respectively.

**Conclusions:**

FCBT is a simple, rapid technique that achieves temporary haemorrhage control in PNI, providing a bridge to definitive surgical control, and may be effective as definitive treatment in selected venous injuries. However, pooled data are limited by heterogeneity in catheter size, balloon inflation and dwell time, restricting interpretability. Standardised multicentre prospective studies are needed to refine indications, optimise technique, and quantify complications.

## Introduction

Penetrating neck injuries (PNI) pose a significant clinical challenge because of the critical density of vascular, aerodigestive and neurological structures in the neck. Uncontrolled haemorrhage remains the leading cause of mortality in PNI. In military settings, up to 21% of preventable battlefield mortality involves junctional haemorrhage in the neck, axilla or groin – regions where standard tourniquets cannot be applied, with similar challenges seen in civilian settings.^1,2^ Traditional control methods, such as direct digital pressure, are ineffective for junctional haemorrhage, necessitating alternative interventions such as wound packing or haemostatic adjuncts, especially when bleeding originates from anatomically inaccessible vascular structures in the neck.^3^

The concept of balloon tamponade was initially adapted from the management of oesophageal variceal bleeding. Foley’s catheter balloon tamponade (FCBT) represents a simple adjunct for temporary haemorrhage control in PNI.^4–6^ The technique involves inserting a Foley catheter through the injury tract and inflating its balloon with fluid until resistance is encountered, effectively providing tamponade for bleeding vessels from in the wound tract, especially in anatomically challenging ‘non-compressible’ areas.^5,7^ This technique was first described by Gilroy *et al* at Baragwanath Hospital in Johannesburg, South Africa, in 1992, and allowed life-threatening exsanguinating haemorrhage to be temporarily controlled pending definitive surgical management.^4^

Since this seminal study, evidence for FCBT in PNI has steadily accumulated; however, most existing studies are limited by small sample sizes, single-centre designs and inconsistent reporting of complications. Consequently, current understanding of the overall efficacy and safety profile of FCBT remains incomplete. To address these gaps, we conducted a systematic review and meta-analysis with the aim of evaluating the efficacy of FCBT for primary haemostasis in PNI, rates of surgical exploration post-FCBT, and associated morbidity and mortality.

## Methods

This review was registered with PROSPERO (CRD420251059225). With reference to the Preferred Reporting Items for Systematic Review and Meta-Analyses (PRISMA) guidelines, a search was conducted on Medline/PubMed, Embase, Cochrane Library and CINAHL databases for studies published before 29 May 2025.^8^ The search strategy used a combination of free text words and Medical Subject Headings terms (Supplementary Text S1). The reference lists of systematic reviews and included articles and the grey literature were also screened manually to identify additional studies, ensuring a comprehensive search. The PRISMA checklist can be found in Supplementary Table S1.

### Study selection

Two reviewers independently screened titles and abstracts to check the eligibility for inclusion using the online platform Rayyan, with disputes being resolved through consensus from a third independent author.^9^ Reviewers then assessed the full texts of shortlisted studies against the following pre-defined inclusion and exclusion criteria.

The inclusion criteria are (1) observational studies that investigated the use of FCBT for active bleeding wounds following a penetrating neck injury, (2) full-text studies, (3) published in a peer-reviewed journal, and (4) published in the English language. The exclusion criteria include (1) animal studies, (2) case reports, (3) in vitro studies and (4) reviews.

### Data extraction

Data from included articles were extracted by two blinded, independent reviewers in duplicate onto a structured proforma specifically designed for the study and piloted beforehand on a sample of selected studies. Disagreement was resolved by discussion and consensus with a third reviewer. Relevant study characteristics and outcome data were extracted on the data extraction spreadsheet, including but not limited to study type, country, sample size, mean age, FCBT success rate, size of catheter used, surgical exploration and mechanism of injury (MOI).

### Quality assessment

Two blinded, independent reviewers conducted the quality assessment of included articles using the Risk Of Bias In Non-randomised Studies – of Interventions (ROBINS-I) tool. The ROBINS-I tool assesses the risk of bias across seven domains, including confounding, selection, intervention classification, deviations from intended interventions, missing data, outcome measurement and selection of reported results.^10^ Each domain and the overall study are rated as either low, moderate, serious or critical risk of bias.

### Statistical analysis

The meta-analysis was conducted using R version 4.2.2 (R Foundation for Statistical Computing), with the package extension meta. Descriptive statistics were presented as means and standard deviations (sd) for continuous variables and counts for categorical variables. When studies reported medians and interquartile ranges, these were converted to means and sd using the methods of Wan *et al*.^11^

For binary outcomes like success after FCBT, rate of FCBT, surgical exploration following successful FCBT, morbidity and mortality, a meta-analysis of proportions was performed using the restricted maximum-likelihood method with the Freeman–Tukey double-arcsine transformation. The rates we report do not reflect population-level prevalence, because only studies reporting FCBT use were included. Statistical heterogeneity was assessed via *I*^2^ and Cochran Q test values, where *I*^2^ < 25% represented low heterogeneity and *I*^2^ ≥ 25% represented moderate to high heterogeneity.^12,13^ A Cochran Q test with a *p*-value of ≤0.10 was considered significant for heterogeneity. Random effects models were used in all analyses regardless of heterogeneity, as published evidence suggests that they provide more robust outcome measures compared with the alternative fixed effects models.^14^ When three or more studies were available, 95% prediction intervals were computed to estimate the potential range of true effect sizes across individual studies, given that the 95% confidence interval (CI) only accounts for the uncertainty of the mean effect size, but not the uncertainty of interstudy variance.^15^

Additional analyses were also conducted to evaluate potential sources of heterogeneity between studies.^16^ Apart from subgroup analyses, univariate random effects meta-regression was conducted, and effect moderators were confirmed using permutation testing with 1,000 iterations to eliminate spurious results.^17,18^ Statistical significance was considered for outcomes with a *p-*value ≤0.05. Publication bias was assessed through visual inspection of the funnel plots, with missing studies imputed using the trim-and-fill method.^19^

### Certainty of evidence

The quality of pooled evidence was evaluated using the Grading of Recommendations Assessment, Development and Evaluations (GRADE) framework.^20^ The GRADE framework rates each study on the basis of study design, consistency, directness, risk of bias, precision and publication bias. For each outcome, the level of evidence was rated as high, moderate, low or very low.

## Findings

### Study and patient characteristics

A total of 155 articles were included in the initial search after removing duplicates, and nine articles were selected for full-text review. All nine articles met the final inclusion criteria (Supplementary Figure S1); two of these studies were prospective cohort studies, and seven studies were retrospective cohort studies.^4,5,7,21–26^ The total sample size was 1,658 patients. The mean (sd) age was 30.99 (7.98) years, and 92.88% of patients were male. Table 1 contains a summary of the key study and patient characteristics. Of the nine included studies, six were at low risk of bias and three were at moderate risk of bias (Supplementary Figure S2).

**Table 1:** Study characteristics

Study	Study type	Country	Sample size	Age (years)	Male (%)	FCBT (%)	Success rate (%)	Size of catheter (Fr)	Volume inflated (ml)	Time at catheter removal (h)	Bleeding on catheter removal (%)
Gilroy *et al*^4^ (1992)	Prospective cohort	South Africa	14	NR	88.00	57.10	62.50	18 or 20	15–20	NR	NR
Himmler *et al*^22^ (2021)	Retrospective cohort	USA	4	31 (8)	100	100	100	18	NR	NR	NR
Jose *et al*^23^ (2019)	Retrospective cohort	India	7	NR	100	100	85.71	18 or 20	10–30	72.00	NR
Kong *et al*^7^ (2022)	Retrospective cohort	South Africa	1,581	NR	93.00	3.00	80.00	18 or 20	5–10	NR	3.70
Madsen *et al*^24^ (2018)	Retrospective cohort	South Africa	817	NR	NR	0.13	72.70	NR	NR	0.50–48.00	72.73
Navsaria *et al*^25^ (2006)	Retrospective cohort	South Africa	220	NR	NR	8.18	94.40	18 or 20	5	48.00–96.00	7.14
Scriba *et al*^5^ (2020)	Retrospective cohort	South Africa	628	NR	NR	15.20	96.80	18 or 20	NR	48.00	2.78
Van Waes *et al*^21^ (2012)	Prospective cohort	South Africa	77	30.75 (7.69)	90.91	9.10	85.70	NR	NR	NR	0.00
Weppner^26^ (2013)	Retrospective cohort	USA	155	26.00 (5.00)	NR	27.10	88.10	18	NR	NR	NR

Mean (sd); FCBT = Foley catheter balloon tamponade; NR = not reported

The Foley catheter sizes used were generally 18 or 20Fr, with balloon inflation volumes varying between 5 and 30ml. The time at catheter removal ranged from 0.50 to 96.00h across the studies. Stab wounds were the most common MOI, followed by gunshot wounds (GSWs); less frequent mechanisms included grenades, improvised explosive devices (IEDs) and rockets (Table 2). Injuries predominantly involved zone II of the neck, followed by zone I and zone III (Table 2). Multiple neck zones were often involved in the same patient.

**Table 2:** Mechanism and location of injury in patients who underwent FCBT (%)

	Mechanism of injury										
Study	Stab wound	GSW	Grenade	IED	Rocket	Zone I	Zone II	Zone III	Multiple zones	Posterior triangle	Supraclavicular fossa
Gilroy *et al*^4^ (1992) (*n* = 8)	62.50	37.50	0.00	0.00	0.00	N	N	N	N	N	Y
Himmler *et al*^22^ (2021) (*n* = 4)	Y	Y	N	N	N	NR	NR	NR	NR	NR	NR
Jose *et al*^23^ (2019) (*n* = 7)	N	Y	Y	N	N	NR	NR	NR	NR	NR	NR
Kong *et al*^7^ (2022) (*n* = 44)	93.00	7.00	0.00	0.00	0.00	50.00	50.00	16.00	16.00	14.00	4.50
Madsen *et al*^24^ (2018) (*n* = 11)	Y	Y	N	N	N	Y	Y	Y	Y	Y	N
Navsaria *et al*^25^ (2006) (*n* = 18)	Y	Y	N	N	N	16.67	50.00	5.56	0.00	2.78	0.00
Scriba *et al*^5^ (2020) (*n* = 95)	Y	Y	N	N	N	22.10	34.70	10.50	14.70	17.90	0.00
Van Waes *et al*^21^ (2012) (*n* = 7)	Y	Y	N	N	N	Y	Y	Y	N	Y	N
Weppner^26^ (2013) (*n* = 42)	0.00	35.70	0.00	57.10	7.20	28.60	42.90	16.70	11.80	0.00	0.00

FCBT = Foley catheter balloon tamponade; GSW = gunshot wound; IED = improvised explosive device; N = no; NR = not reported; Y = yes

The types of vascular injuries were diverse, encompassing arterial, venous and mixed injuries. Frequently injured vessels included major structures such as the common carotid artery (CCA), external carotid artery (ECA), internal jugular vein (IJV), subclavian vessels and various unspecified cervical arteries and veins. Notably, several studies reported mixed arterial and venous injuries or unspecified vascular injuries (Table 3).

**Table 3:** Vascular injury in patients who underwent FCBT (%)

Study	CCA	ECA	ICA	SA	BA	CT	VA	TT	TA	LA	FA	SSA	Unspecified arteries	SV	IJV	EJV	RV	BV	Left BV/IV	Unspecified veins	Unspecified vessel(s)	Mixed arterial and venous	Equivocal CTA venous findings	No VI
Gilroy *et al*^4^ (1992) (*n* = 8)	0.00	0.00	0.00	0.00	0.00	0.00	0.00	0.00	0.00	0.00	0.00	0.00	0.00	25.00	25.00	0.00	0.00	0.00	0.00	0.00	0.00	50.00	0.00	0.00
Himmler *et al*^22^ (2021) (*n* = 4)	0.00	0.00	0.00	0.00	0.00	0.00	0.00	0.00	0.00	0.00	0.00	0.00	0.00	50.00	0.00	0.00	0.00	0.00	25.00	0.00	0.00	25.00	0.00	0.00
Jose *et al*^23^ (2019) (*n* = 7)	N	Y	N	N	N	N	N	N	N	N	N	N	NR	N	Y	N	N	N	N	N	N	N	N	N
Kong *et al*^7^ (2022) (*n* = 44)	5.00	2.00	0.00	2.00	0.00	2.00	2.00	0.00	0.00	0.00	0.00	0.00	0.00	0.00	5.00	0.00	0.00	0.00	0.02	0.00	0.00	2.00	0.00	0.00
Madsen *et al*^24^ (2018) (*n* = 11)	0.00	0.00	0.00	0.00	0.00	0.00	0.00	0.00	0.00	0.00	0.00	0.00	0.00	9.00	36.00	18.00	0.00	0.00	0.00	0.00	0.00	45.00	0.00	0.00
Navsaria *et al*^25^ (2006) (*n* = 18)	Y	N	N	Y	N	N	N	Y	N	N	N	N	N	N	Y	N	N	N	N	N	N	N	N	N
Scriba *et al*^5^ (2020) (*n* = 95)	8.00	1.00	1.00	4.00	2.00	0.00	3.00	4.00	3.00	2.00	1.00	1.00	4.00	2.00	14.00	4.00	NR	2.00	0.00	0.05	0.00	0.13	16.00	42.00
Van Waes *et al*^21^ (2012) (*n* = 7)	14.00	0.00	14.00	29.00	0.00	0.00	14.00	0.00	0.00	0.00	0.00	0.00	0.00	0.00	0.00	0.00	0.00	0.00	0.00	0.00	0.00	0.00	0.00	0.00
Weppner^26^ (2013) (*n* = 42)	12.00	24.00	12.00	10.00	0.00	0.00	19.00	0.00	0.00	0.00	0.00	0.00	0.00	14.00	12.00	7.00	0.00	0.00	0.00	0.00	43.00	0.00	0.00	0.00

BA = brachiocephalic artery; BV = brachiocephalic vein; CCA = common carotid artery; CT = costocervical trunk; ECA = external carotid artery; EJV = external jugular vein; FA = facial arteries; FCBT = Foley catheter balloon tamponade; ICA = internal carotid artery; IJV = internal jugular vein; IV = innominate vein; LA = lingual arteries; N = no; NR = not reported; PNI = penetrating neck injury; RV = retromandibular vein; SA = subclavian artery; SSA = suprascapular arteries; SV = subclavian vein; TT = thyrocervical trunk; TA = thyroid arteries; VA = vertebral artery; VI = vascular injury; Y = yes

### Meta-analysis of the rate of FCBT use and FCBT success rate

**Figure 1:**
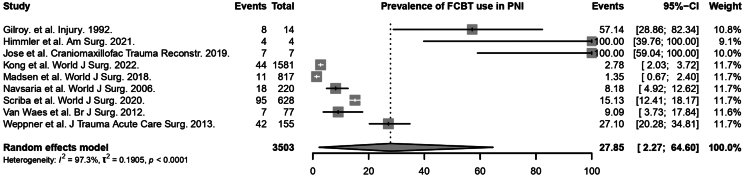
Rate of Foley catheter balloon tamponade use in penetrating neck injuries

The rate of FCBT use was reported in all 9 studies (236 patients).^4,5,7,21–26^ Based on the random effects model, the rate of FCBT use in patients with actively bleeding penetrating neck injury was significant at 27.85% (95% CI 2.27 to 64.60, *I*^2^ = 97%, *p* < 0.01) (Figure 1). Results of subgroup analyses can also be found in Supplementary Table S2. It was found that there were significant differences in the pooled rate of FCBT use across subgroups of geographical region (*p* < 0.01). There was one study based in Asia, two studies in the United States of America (USA) and six studies in Africa. The rate of FCBT use was 100% in Asia, 63.81% in the USA and 10.28% in Africa. However, there were no significant differences observed across the MOI subgroups (*p* = 0.2689).

Meta-regression found that study-level characteristics, including mean age and gender (male = 92.88%), were not significant effect moderators of the rate of FCBT use among this group of patients. The results of the meta-regression are shown in Supplementary Table S3.

**Figure 2:**
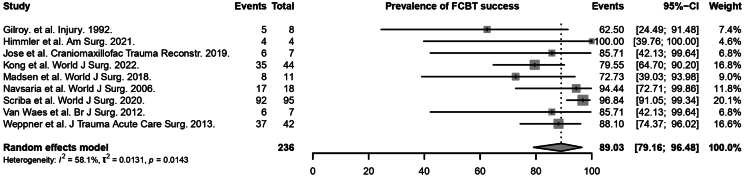
Rate of Foley catheter balloon tamponade success

The success rate of achieving primary haemostasis following FCBT insertion was also reported in all 9 studies (210 patients).^4,5,7,21–26^ It ranged from 62.50% to 100% across studies, with most studies reporting success rates exceeding 80.00% (Table 1). Based on the random effects model, the rate of FCBT success was significant at 89.03% (95% CI 79.16 to 96.48, *I*^2^ = 58%, *p* = 0.0143) (Figure 2). Results of subgroup analyses can also be found in Supplementary Table S2. There were no significant differences observed between subgroups of geographical region (*p* = 0.7480), size of catheter used (*p* = 0.7662), volume inflated into balloon (*p* = 0.2690) and duration before removal of catheter (*p* = 0.8902). However, there were significant differences in the pooled rate of FCBT success across subgroups of different MOI (*p* < 0.01). Seven studies reported MOI of stab wounds and/or GSW, and two studies reported MOI of GSW and/or others like grenade, IED or rocket (Table 2). The rate of FCBT success was 88.67% among patients with stab wounds and/or GSW, and 89.24% among patients with GSW and/or other MOIs.

Meta-regression found that mean age and gender (male = 92.88%) were also not significant effect moderators of the rate of FCBT success among this group of patients. The results of the meta-regression are shown in Supplementary Table S3.

### Surgical exploration and rebleeding outcomes

**Figure 3:**
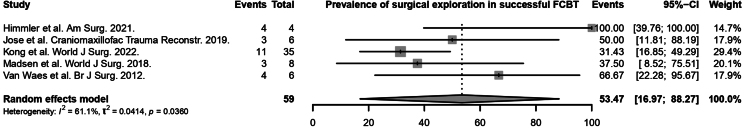
Rate of surgical exploration among successful Foley catheter balloon tamponade

Surgical exploration rates following FCBT varied significantly, ranging from 31.43% to 100% of cases across studies (Table 4). The primary indications for surgical exploration included major vascular injuries and rebleeding upon catheter removal. Of the nine studies, only five (25 patients) reported the rate of surgical exploration following FCBT success.^7,21–24^ Based on a random effects model, the rate of surgical exploration was significant at 53.47% (95% CI 16.97 to 88.27, *I*^2^ = 61%, *p* = 0.0360) (Figure 3). Subgroup analyses on geographical region, MOI and indications for surgical exploration were also performed, and results of the analyses can be found in Supplementary Table S2. There were no significant differences in the pooled rate of surgical exploration among successful FCBT across subgroups of MOI (*p* = 0.8310). However, there were significant differences across subgroups of geographical region (*p* = 0.0277) and indications for surgical exploration (*p* < 0.01). For the subgroup analyses of geographical region, one study was done in Asia, one was done in the USA and three were done in Africa. The rate of surgical exploration among successful FCBT was 50.00% is Asia, 100% in the USA and 37.41% in Africa. For the subgroup analyses of indications for surgical exploration, two studies reported major vascular injury only, and two other studies reported major vascular injury or rebleeding on catheter removal. It was found that the rate of surgical exploration was 58.43% in the subgroup of major vascular injury only, whereas the rate of surgical exploration was lower at 32.01% in the subgroup of patients with major vascular injury or rebleeding on catheter removal.

**Table 4:** Management outcomes following successful FCBT in PNI (%)

		Reason for surgical exploration	
Study	Surgical exploration	Major vascular injury	Rebleeding on catheter removal
Gilroy *et al*^4^ (1992) (*n* = 5)	NR	NR	NR
Himmler *et al*^22^ (2021) (*n* = 4)	100	NR	NR
Jose *et al*^23^ (2019) (*n* = 6)	50.00	50.00	0.00
Kong *et al*^7^ (2022) (*n* = 35)	31.43	31.43	2.86
Madsen *et al*^24^ (2018) (*n* = 8)	37.50	37.50	62.50
Navsaria *et al*^25^ (2006) (*n* = 17)	NR	NR	5.88
Scriba *et al*^5^ (2020) (*n* = 92)	NR	NR	2.17
Van Waes *et al*^21^ (2012) (*n* = 6)	66.67	66.67	0.00
Weppner^26^ *(*2013) (*n* = 37)	NR	NR	NR

FCBT = Foley catheter balloon tamponade; NR = not reported; PNI = penetrating neck injury

Meta-regression found that gender (male = 92.88%) was also not a significant moderator of the rate of surgical exploration among patients with successful FCBT. Meta-regression for mean age was not performed due to a lack of data. The results of the meta-regression are shown in Supplementary Table S3.

### Morbidity and mortality

**Figure 4:**
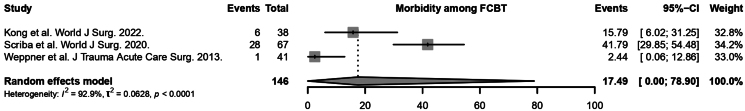
Morbidity among successful Foley catheter balloon tamponade

There was a lack of reported complications following FCBT; however, in those studies that reported them, morbidity rates were 2.38% for Weppner *et al*, 14.00% for Kong *et al* and 29.47% for Scriba *et al* (Table 5). The rate of morbidity was reported in three studies (35 patients).^5,7,26^ Based on a random effects model, the rate of morbidity among PNI patients with FCBT was significant at 17.49% (95% CI 0.00 to 78.90, *I*^2^ = 93%, *p* < 0.01) (Figure 4).

**Table 5:** Morbidity and mortality following FCBT in PNI (%)

Study	Morbidity	Mortality	Respiratory complications	ICU admission	Pressure ulcers	Wound site infection	CVA or stroke	Pneumonia/Empyema	Massive blood transfusion	Cardiac arrest needing CPR
Gilroy *et al*^4^ (1992) (*n* = 8)	NR	NR	NR	NR	NR	NR	NR	NR	NR	NR
Himmler *et al*^22^ (2021) (*n* = 4)	NR	NR	NR	NR	NR	NR	NR	NR	NR	NR
Jose *et al*^23^ (2019) (*n* = 7)	NR	NR	NR	NR	NR	NR	NR	NR	NR	NR
Kong *et al*^7^ (2022) (*n* = 44)	14.00	11.00	7.00	18.00	2.00	NR	NR	NR	NR	NR
Madsen *et al*^24^ (2018) (*n* = 11)	NR	NR	NR	NR	NR	NR	NR	NR	NR	NR
Navsaria *et al*^25^ (2006) (*n* = 18)	NR	NR	NR	NR	NR	NR	NR	NR	NR	NR
Scriba *et al*^5^ (2020) (*n* = 95)	29.47	4.20	27.37	15.80	NR	8.42	5.26	5.26	4.21	4.21
Van Waes *et al*^21^ (2012) (*n* = 7)	NR	NR	NR	NR	NR	NR	NR	NR	NR	NR
Weppner^26^ (2013) (*n* = 42)	2.38	4.76	NR	NR	NR	NR	NR	NR	NR	NR

CPR = cardiopulmonary resuscitation; CVA = cerebrovascular accident; FCBT = Foley catheter balloon tamponade; ICU = intensive care unit; NR = not reported; PNI = penetrating neck injury

**Figure 5:**
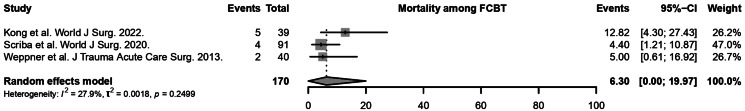
Mortality among successful Foley catheter balloon tamponade

The rate of mortality was reported in three studies (11 patients).^5,7,26^ Mortality rates ranged from 2.38% for Weppner *et al* to 14.00% for Kong *et al* and 29.47% for Scriba *et al* (Table 5). Based on a random effects model, the rate of mortality among PNI patients with FCBT was not significant at 6.30% (95% CI 0.00 to 19.97, *I*^2^ = 28%, *p* = 0.2499) (Figure 5).

## Discussion

This systematic review of 9 studies, encompassing 1,658 patients provides evidence supporting the efficacy of FCBT in achieving primary haemostatic control following PNI. The pooled success rate of 88.67% (95% CI 74.20 to 98.37) among patients with stab wounds and/or GSW, and 89.24% (95% CI 56.33 to 100) among patients with GSW and/or other MOIs highlights FCBT as a useful adjunct.

In most series, FCBT was deployed in the resuscitation bay as a temporising manoeuvre for unstable bleeding. Once haemostasis and physiology improved, patients typically underwent computed tomography angiography or formal catheter angiography to exclude a named-vessel injury. Negative imaging or minor venous/arterial injuries were managed by leaving the catheter *in situ*, with patients observed in a high-dependency unit for 24 to 72h before elective removal under controlled conditions.^7,21,24,25^ Conversely, just over half of the tamponaded patients subsequently underwent operative or endovascular intervention – predominantly for major arterial injuries or rebleeding at supervised balloon removal. This staged pathway mirrors trauma training and guidance. Western Trauma Association recommendations emphasise immediate life-saving haemorrhage control followed by selective imaging and tailored definitive management for penetrating neck trauma, recognising tamponade as a valid temporising step for junctional bleeding.^27^ Contemporary reviews likewise describe FCBT as an accepted technique for rapid haemostasis pending definitive surgical or endovascular control.^28,29^ In combat settings, FCBT has been associated with improved survival in penetrating neck or maxillofacial injuries compared with direct pressure, supporting its role as a bridge to definitive care.^26^

The relationship between injury severity, specifically major vascular involvement, and the subsequent need for surgical exploration was notable. Pooled data demonstrated that surgical exploration occurred in 58.43% (95% CI 0.00 to 100) of patients with major vascular injuries vs 32.01% (95% CI 3.43 to 69.96) when exploration was triggered by rebleeding at removal. Together with our meta-regression, this highlights the influence of vessel calibre on clinical decision making, reinforcing the notion that major vascular disruptions – particularly involving high-pressure vessels like the CCA, subclavian artery or vertebral artery – necessitate surgical exploration and are more prone to rebleeding on removal. Series that used larger fill volumes or multiple balloons where necessary reported fewer failures, supporting a pressure/footprint threshold phenomenon.^5^ Collectively, our results and existing literature indicate that proximity to, or disruption of, high-calibre vessels should lower the threshold for early operative or endovascular control.

Although some series have demonstrated the potential of FCBT as a form of definitive management (without the need for ligation or surgical repair) in selected cases of minor arterial or venous injuries in the neck, our meta-analysis demonstrated that more than 50% of patients who achieved primary haemostasis with FCBT still proceeded to surgical exploration.^4,5,24,25^ Patient selection (e.g. isolated venous trauma and hemodynamic stability) and adherence to an appropriate dwell time before trial removal of the balloon are essential, because premature deflation can lead to failure.^24^ These observations temper the enthusiasm for FCBT as a stand-alone therapy and universally curative measure. Given small sample sizes, retrospective designs and methodological heterogeneity, our pooled estimates should be regarded as hypothesis-generating rather than definitive. Catheter size, balloon inflation volume and dwell time varied widely, limiting interpretability and the statistical power of subgroup analyses. This challenge of heterogeneity is well recognised in trauma meta-analyses, particularly in interventions with variable technical parameters.

Stab wounds accounted for most cases reported, followed by GSWs and blast injuries. Of note, several papers collapsed GSWs into both MOI strata, forcing us to dichotomise studies as ‘stab ± GSW’ vs ‘GSW ± other MOI’ and leaving pure-GSW data unanalysed. Higher-energy trauma, such as GSW, IEDs or grenade injuries, typically present with more complex injury patterns, including multizone cavitating wounds that exceed the compressive capability of a balloon catheter, which might lead to lower success rates.^28,29^ In the presence of more complex injuries, other management strategies may be required. Prospective registries that separate MOI more granularly (e.g. stab wounds, GSWs, blast injuries) are necessary to clarify whether energy transfer, tissue devitalisation or wound geometry best predicts FCBT efficacy.

Anatomical site also influenced outcomes. Zone II injuries predominated, consistent with civilian trauma epidemiology, because this zone is more readily accessible and amenable to external interventions.^30,31^ Historically, injuries in zones I or III are considered more lethal because of a higher density of vital structures and poorer surgical access.^28,32,33^ Balloon seating against rigid structures (e.g. mandible, vertebral bodies) might also influence the success rate of achieving primary haemostasis.

Surgical specialty represents another contextual factor. In many trauma centres, FCBT is deployed by general or trauma surgeons, whereas in other settings, the initial responders may be otorhinolaryngologists or maxillofacial or plastic surgeons with variable familiarity with junctional vascular control. This variability in training and exposure could influence the decision to use FCBT and subsequent pathways to definitive repair. Although our review did not stratify outcomes by surgeon type, future studies should investigate this factor, particularly in resource-limited settings that lack immediate access to vascular or trauma-trained surgeons.

Overall morbidity and mortality were low, but only three studies reported complications, and none systematically captured balloon dwell time adverse events (e.g. airway compression or neurovascular compromise). Future studies should adopt uniform definitions and, where possible, follow-up imaging to accurately characterise risks and refine safety protocols. Nevertheless, observed complication rates compared favourably with historical series of blind wound packing or immediate exploration, reinforcing the safety profile of FCBT.^34–36^

Future research should move beyond retrospective single-centre series towards multicentre registries and comparative trials to clearly define the role of FCBT in trauma algorithms. Research priorities include: (1) standardisation of catheter characteristics (e.g. size, inflation volume, dwell time); (2) stratifying outcomes by MOI and vessel type; (3) prospective reporting of complications such as airway compression and neurovascular compromise; and (4) comparative effectiveness studies evaluating FCBT against wound packing, haemostatic dressings, and endovascular balloon devices.

Our review has several limitations. The small number of studies and limited data from specific geographical regions (notably Asia and the USA) restricts the representativeness of reported rates of FCBT use. Differences in reported rates of FCBT use between regions likely reflect disparities in trauma system maturity, resource availability and clinician familiarity rather than true clinical variation. In high-resource settings such as the USA, ready access to hybrid operating suites, vascular surgeons and endovascular devices often allows immediate definitive repair without prolonged temporising measures. Conversely, in resource-limited environments, general surgeons and emergency physicians may rely more heavily on simple mechanical methods such as FCBT to achieve temporary haemostasis when theatre access, interventional radiology or blood products are limited. These contextual differences underscore that the rate of FCBT use in our analysis reflects practice patterns in available infrastructure rather than a direct epidemiological indicator. In addition, the pooled cohort was predominantly male (92.88%), with six of nine studies included originating from South Africa; consequently, the findings may not be fully generalisable to other demographics or clinical settings that have different pre-hospital and surgical resources. Also, reporting was heterogeneous (e.g. catheter size and balloon inflation volume) as described above, which may reduce the statistical power of subgroup analyses. Finally, the way in which MOI was reported hampered a clean stab vs other comparison.

Clinically, our findings support the role of FCBT in broader junctional haemorrhage management algorithms. It appears effective in achieving temporary control of life-threatening haemorrhage from PNI, serving as a bridge to definitive surgical management. Furthermore, it may serve as the definitive treatment in a selected group of patients, especially for venous injuries.

## Conclusion

Our review synthesises the largest body of evidence to date on FCBT in the control of life-threatening haemorrhage from PNI. FCBT shows promise as a temporising measure in penetrating neck trauma, but current evidence is limited by heterogeneity and methodological constraints. Our pooled findings should be interpreted as hypothesis-generating signals rather than definitive figures. FCBT provides valuable time for airway control, imaging and surgical exploration. In high-energy mechanisms and major arterial disruption, immediate surgical or endovascular management remains essential. Standardised protocols and prospective registries will be critical to refining indications, minimising complications, and optimising outcomes for this simple yet life-saving technique.

## Data Availability

All data underlying this article are from previously published studies, which are available in Medline/PubMed, Embase, the Cochrane Library, and CINAHL.

## Artificial Intelligence

The author/s declare that no AI was used to conduct the study or prepare the manuscript.
